# The Role of Psychosocial Risk Status in Relation to Outcomes Following Cognitive Behavioral Therapy for Youth with Abdominal Pain-Related Disorders of Gut–Brain Interaction

**DOI:** 10.1007/s10880-026-10151-2

**Published:** 2026-04-20

**Authors:** Thea Senger-Carpenter, Steven Pierce, Jocelyn Zuckerman, Elise Cheney-Makens, Michelle Adler, Natoshia Cunningham

**Affiliations:** 1 College of Human Medicine, Department of Family Medicine, Michigan State University, East Lansing, Michigan USA; 2 Henry Ford Health, Detroit, MI United States; 3 Center for Statistical Training and Consulting, Michigan State University, East Lansing, Michigan USA; 4 College of Human Medicine, Michigan State University, East Lansing, Michigan USA

**Keywords:** Abdominal pain, Pediatric, Youth, Cognitive behavioral, Psychosocial

## Abstract

It is unknown whether youth with abdominal pain-related disorders of gut–brain interaction (AP-DGBI) and clinically elevated pain intensity, functional disability, and anxiety (“high-risk”) respond differentially to the Aim to Decrease Anxiety and Pain Treatment (ADAPT), an evidence-based cognitive behavioral therapy program, compared to youth with fewer risk factors (“low-risk”). This secondary analysis included youth aged 9–14 with AP-DGBI recruited from outpatient gastroenterology clinics and randomized to receive ADAPT plus treatment as usual (TAU) or TAU-alone. Baseline risk status was determined using an established grading system derived from validated measures. Differences in post-treatment (~ 8 weeks) pain intensity, functional disability, and anxiety were examined using a multivariate analysis of covariance model with an interaction of risk-status-by-treatment-allocation. Data from 79 youth were analyzed; 29 (36.7%) were high-risk. Risk status (F(3, 72) = 3.30, Wilks’ Λ = 0.879, *p* = 0.025) predicted higher post-treatment pain intensity (estimated marginal mean difference high versus low risk 1.47 [95% Confidence Interval (CI) 0.44, 2.50]) and functional disability (5.71 [95% CI 1.38, 10.05]), but not anxiety. Accounting for risk status, ADAPT did not affect post-treatment outcomes and no risk-by-treatment-interaction emerged. Risk status predicted post-treatment outcomes for youth with AP-DGBI. Clinical implications include screening of psychosocial risk factors.

## Introduction

Nearly 12% of youth worldwide are affected by abdominal pain-related disorders of gut–brain interaction (AP-DGBI) (Campo et al., 2002; Vermeijden et al., 2025; Zia et al., 2022). Also referred to as functional abdominal pain disorders, AP-DGBI are a set of conditions characterized by recurrent abdominal pain occurring in the absence of an identifiable structural or biochemical abnormality (Fairlie et al., 2023; Rurgo et al., 2024). AP-DGBI have been associated with a lower quality of life, increased healthcare spending, and functional disability (Hoekman et al., 2015; Thapar et al., 2020). While the etiology of AP-DGBI is not fully understood, they are widely acknowledged to arise from, and be sustained by, interacting biological (e.g., genetic predisposition, inflammation, differences in motility and gut microbiota) and psychosocial (stress, coping styles, parent behaviors) factors (Drossman, 2016; Thapar et al., 2020).

While AP-DGBI symptoms resolve for some, youth with co-occurring clinically elevated pain, functional disability, and anxiety symptoms (based on established cut-points for validated symptom measures that typically indicate significantly impaired functioning and/or wellbeing) may be likelier to experience persistent impairment (Cunningham et al., 2017). Youth with all three of these psychosocial risk factors present have also exhibited sub-clinically elevated fecal calprotectin (a biomarker for gastrointestinal inflammation) (Moorman et al., 2021), suggesting a “high-risk” AP-DGBI subgroup characterized by greater biopsychosocial complexity. Further, youth with elevated pain and functional disability may be at increased risk for persistent AP-DGBI symptoms into adolescence and young adulthood (Walker et al., 2012). Given the negative impacts of AP-DGBI on social as well as physical and mental adult health outcomes, (Horst et al., 2014; Shelby et al., 2013; Stone et al.,^ 2023^; Walker et al., 2012), prompt, effective intervention may be especially critical for this high-risk subgroup.

It has already been shown that clinically elevated anxiety may impair treatment response to cognitive behavioral therapy (CBT) among youth with chronic pain conditions including AP-DGBI (Cunningham et al., 2016). It is unknown how being “high-risk” (e.g., evidencing clinically elevated pain, functional disability, and anxiety symptoms based on scores derived from valid, symptom-specific measures) impacts response to CBT, which is considered a central component of AP-DGBI management (Rutten et al., 2015; Thapar et al., 2020). Indeed, previous work developing the Aim to Decrease Anxiety and Pain Treatment (ADAPT) program—a tailored CBT program addressing anxiety and pain—demonstrated efficacy for both pain-related functional disability and anxiety symptoms in youth with AP-DGBI (Cunningham et al., 2018a, 2018b, 2021). Understanding whether youth with co-occurring clinically elevated pain, functional disability, and anxiety (e.g., “high-risk” AP-DGBI) at baseline respond differentially to ADAPT compared to youth with fewer risk factors (“low- risk”) may be salient to refining approaches to care. This study, therefore, compares the impact of ADAPT on post-treatment average pain intensity, functional disability, and anxiety symptoms for youth with high- versus low-risk AP-DGBI.

## Methods

### Design

This is a secondary analysis of data from a randomized controlled trial (RCT) conducted by the senior author from July 20, 2015 to August 2, 2018, which examined the efficacy of ADAPT and the feasibility of a stepped approach to care (Cunningham et al., 2021). Participants in the original study were youth aged 9–14 years diagnosed with AP-DGBI by a gastroenterologist using Rome IV criteria (Koppen et al., 2017), recruited from the outpatient pediatric gastroenterology clinics of a major Midwestern children’s hospital (Cunningham et al., 2021). Eligibility was further determined by greater than minimal functional disability (see Measures), while exclusion criteria were limited to significant medical conditions including those associated with abdominal pain (e.g., inflammatory bowel diseases), severe depression (i.e., a Childhood Depression Inventory Second Edition [CDI 2] T-score > 80), active suicidal ideation, or other severe psychiatric concerns limiting youths’ ability to engage with tailored CBT. The current analyses were conducted using data from youth randomized to ADAPT plus treatment as usual (TAU) or TAU-alone with complete post-treatment assessment data.

## Procedure

The parent RCT is detailed extensively elsewhere (Cunningham et al., 2021). Briefly, all enrolled participants (n = 139) received pain-focused psychoeducation and relaxation skills training during a gastroenterology visit and were provided with access to online materials supporting home practice. Those who did not evidence a reduction in functional disability to no more than minimal levels (i.e., Functional Disability Inventory [FDI] scores ≤ 7) two weeks later were randomized on a 1:1 basis to receive ADAPT plus TAU or TAU-alone (n = 89; 44 in ADAPT + TAU, 45 in TAU-alone). Notably, TAU comprised evidence-based biomedical management strategies, which were tailored to the individual patient and may have included pharmacotherapy (e.g., antispasmodic agents, acid reduction therapies), recommendations for diet modification, and a referral for CBT (Cunningham et al., 2021). Importantly, study staff ensured that there was no overlap between ADAPT and clinical CBT.

Delivery of ADAPT was stratified based on youths’ presentation. Youth *without* clinical anxiety symptoms (based on Screen for Child Anxiety-Related Disorders [SCARED] scores < 25, see Measures) received four weekly sessions of pain-focused CBT from a clinical psychologist (two in person, two via web modules with telephone support). Those *with* clinical anxiety symptoms (based on SCARED scores ≥ 25 or presence of an anxiety disorder determined using the clinician-administered Anxiety Disorder Interview Schedule for DSM-IV, Child Version [ADIS-IV-C]) (Silverman et al., 2001) received six weekly sessions of CBT that addressed both pain and anxiety symptoms (two in person, four web-based sessions with telephone support). Session content included psychoeducation, cognitive (e.g., thought restructuring), and behavioral (e.g., relaxation training, activity pacing) strategies, as well as mindfulness meditation approaches (Cunningham et al., 2018a, 2018b). Treatment integrity was assessed using standardized fidelity checklists (range 0–100%) measuring providers’ adherence to the ADAPT treatment protocol, completed by independent reviewers. Findings from the parent study indicated high levels of integrity across sessions (mean 98%, range 88–100%) (Cunningham et al., 2021).

## Measures

### Exposure Variable

*AP-DGBI Risk Status.* Youth with clinically elevated pain intensity, functional disability, and anxiety symptoms at baseline were classified as having high-risk AP-DGBI, as per earlier work (Cunningham et al., 2017). Clinical elevation is defined for each symptom below. Youth with zero, one, or two clinically elevated scores were classified with low-risk AP-DGBI, based on data indicating that youth with fewer than three risk factors were less likely to evidence elevated fecal calprotectin suggestive of a high-risk AP-DGBI phenotype (Moorman et al., 2021).

### Outcomes

*Pain Intensity*: Youth reported their past two-week average pain intensity at baseline and post-treatment (~ 8 weeks later) using an eleven-point visual analog scale (VAS), anchored by “no pain” (0) and “worst pain” (10) (McGrath et al., 2008). This approach has been validated for children and adolescents (Stinson et al., 2006), with ratings ≥ 4 suggesting clinically elevated pain intensity, and reductions of ≥ 30% indicating clinically meaningful improvement (Castarlenas et al., 2017; Farrar et al., 2001; Voepel-Lewis et al., 2011).

*Functional Disability*: Pain-related functional disability was assessed at baseline (α = .85) and post-treatment (*α* = .89) using the child-reported Functional Disability Inventory (FDI), a valid and reliable 15-item measure of perceived difficulty performing tasks across home, school, recreational, and social settings over the past few days (Cunningham et al., 2014; Kashikar-Zuck et al., 2011; Walker & Greene, 1991). Items such as “being awake all day without a nap/rest” were rated from 0 (“no trouble”) to 4 (“impossible”) and summed, yielding total scores of 0–60 with higher scores indicating greater disability (Walker & Greene, 1991). Total scores ≥ 13 indicate moderate or higher levels of disability and were used to identify clinically elevated symptoms (Cunningham et al., 2021; Sil et al., 2014; Walker & Greene, 1991). A score reduction of ≥ 7.8 points is considered clinically meaningful (Sil et al., 2014).

*Anxiety*: Anxiety symptoms were measured at baseline ( α = .94) and post-treatment (α = .95) using the child-reported Screen for Child Anxiety -Related Disorders (SCARED), a 41-item measure of past three-month symptoms (Birmaher et al., 1997, 1999; Cunningham et al., 2019). Total scores range from 0 to 82, with higher scores suggesting greater anxiety and scores ≥ 25 indicating clinical significance (Birmaher et al., 1999; Caporino et al., 2017). Notably SCARED scores ≥ 25 correlate with, and may serve as a proxy for, anxiety disorder diagnoses among youth with AP-DGBI (Aggarwal Dutta et al., 2021). A 50% reduction in post-treatment SCARED scores is considered clinically meaningful (Caporino et al., 2017).

### Covariates

*Demographics*: Youth’s age in years, sex at birth, racial identity, and medication/supplement usage were reported by caregivers and derived from electronic medical records. Though medication data are not reported here, the parent study noted no differences between the ADAPT + TAU and TAU-alone groups regarding type or number of medications/supplements (Cunningham et al., 2021).

## Analyses

Participant characteristics and post-treatment outcomes were examined for the entire analytic sample, high- and low- AP-DGBI risk status groups. Rates of attrition were compared for the treatment allocation (ADAPT + TAU versus TAU-alone) and risk status groups (high versus low) using chi squared tests. Average baseline symptoms (i.e., pain intensity, functional disability, anxiety) and demographic composition (i.e., sex, racial identity, age) were compared for participants with complete versus missing post-treatment data using chi squared and t-tests, based on variable type.

Differences in the impact of ADAPT on post-treatment pain intensity, functional disability, and anxiety symptoms for high- and low- risk participants were examined using a single multivariate analysis of covariance (MANCOVA) model with an interaction of risk status (high versus low) and treatment allocation (ADAPT + TAU versus TAU-alone) and adjusting for age, in line with earlier related work (Cunningham et al., 2017). Estimated marginal means and mean differences were computed post hoc for significant comparisons. All analyses were conducted using Stata SE version 19.

## Results

The analytic sample included 79 youth with available post-treatment data (age 11.7 years ± 1.8), 29 of whom (36.7%) were classified with high-risk AP-DGBI. The distribution of high- versus low- risk was unrelated to treatment allocation (Table 1), as expected given random assignment. There were no significant differences in rates of missing post-treatment data based on risk status or treatment allocation. Similarly, there were no significant differences in baseline pain intensity, functional disability, anxiety, or demographic composition for youth with complete versus incomplete post-treatment data. Missing data were therefore deemed missing completely at random and addressed using listwise deletion.

**Table 1:** Sample Characteristics, Baseline and Post-treatment Factors

Characteristic	Whole sample (*n* = 79)	High-risk AP-DGBI (*n* = 29)	Low-risk AP-DGBI (*n* = 50)	*p*-value
ADAPT	40 (50.6)	15 (51.7)	25 (50.0)	0.883
Age (years)	11.7 ± 1.8	12.2 ± 1.6	11.4 ± 1.8	0.048
Female sex (male)	47 (59.5)	20 (69.0)	27 (54.0)	0.192
Race	–	–	–	0.874
African American/Black	3 (3.8)	1 (3.5)	2 (4.0)	
Other	4 (5.1)	1 (3.5)	3 (6.0)	
White	72 (91.1)	27 (93.1)	45 (90.0)	
**Baseline factors**				
Baseline average pain intensity (0–10)	3.7 ± 1.8	5.1 ± 1.0	2.9 ± 1.7	N/A
Clinically significant (≥ 4)	41 (51.9)	29 (–)	12 (24.0)	N/A
Baseline functional disability (0–60)	20.4 ± 8.7	24.8 ± 8.2	17.9 ± 8.0	N/A
Clinically significant (≥ 13)	64 (81.0)	29 (–)	35 (70.0)	N/A
Baseline anxiety (0–82)	35.1 ± 15.7	42.5 ± 12.3	30.7 ± 15.9	N/A
Clinically significant (≥ 25)	58 (73.4)	29 (–)	29 (58.0)	N/A
**Post-treatment factors**				
Post-treatment average pain intensity	2.8 ± 2.2	3.6 ± 2.4	2.3 ± 2.0	0.011
Clinically significant	24 (30.4)	13 (44.8)	11 (22.0)	0.033
Post-treatment functional disability	11.6 ± 9.4	14.9 ± 10.6	9.6 ± 8.1	0.014
Clinically significant	31 (39.2)	17 (58.6)	14 (28.0)	0.007
Post-treatment anxiety	25.9 ± 17.0	30.5 ± 17.3	23.3 ± 16.3	0.066
Clinically significant	37 (46.8)	17 (58.6)	20 (40.0)	0.110

*AP-DGBI* abdominal pain-related disorders of gut–brain interaction, *ADAPT* Aim To Decrease Anxiety and Pain Treatment, *TAU* treatment as usual

Data are presented as n(%) or mean ± standard deviation

*p*-values derived from chi squared or t-test comparisons of the high- and low- risk groups. Statistical comparison is not appropriate for baseline factors, as these were used to define the high- and low- risk groups; *p*-values for these comparisons are therefore marked N/A

**Fig. 1:**
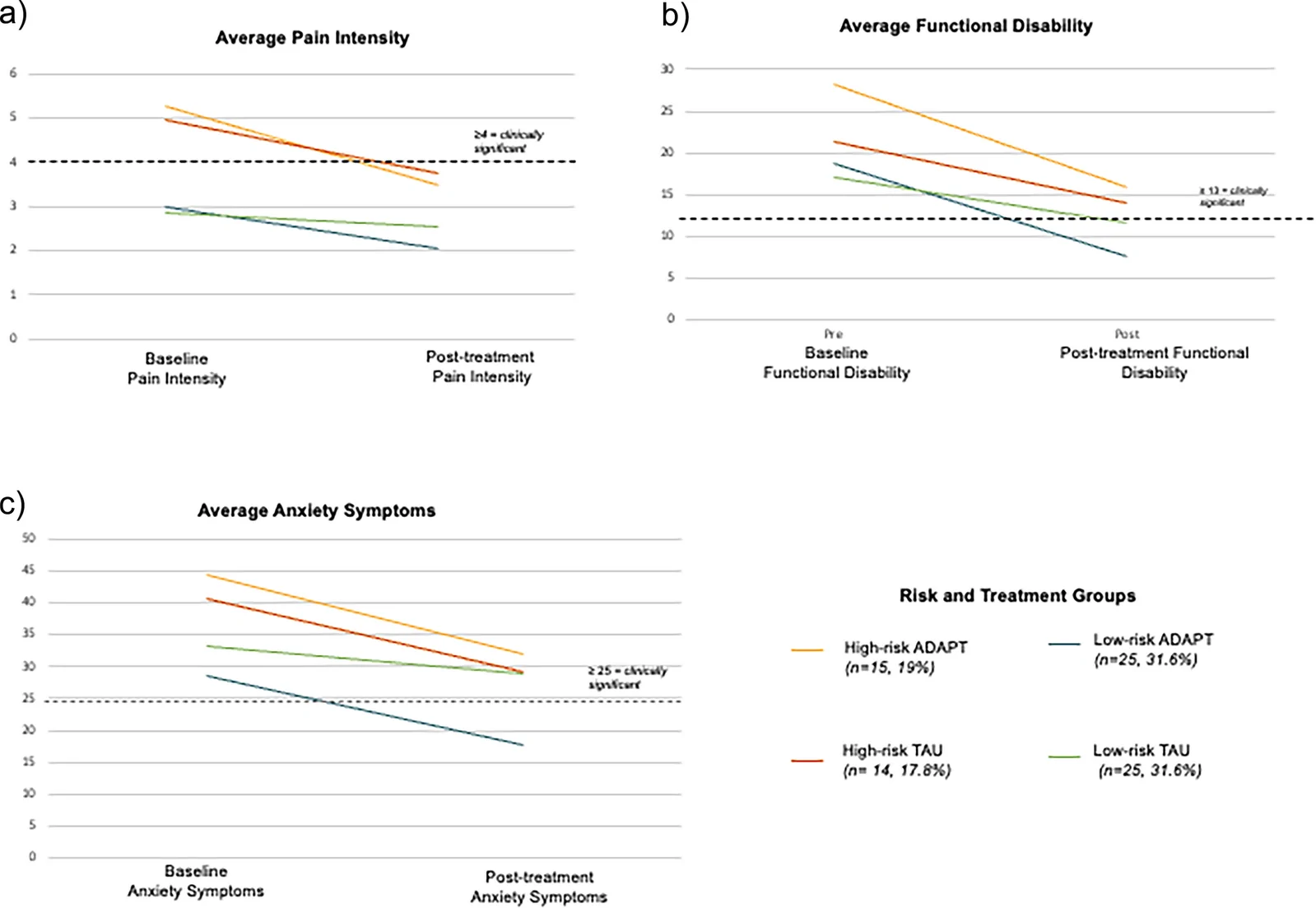
Average raw (versus model-derived) pain intensity, functional disability, and anxiety symptoms from pre- to post-treatment based on high- and low- abdominal pain-related disorders of gut–brain interaction (AP-DGBI) risk status and treatment allocation

The entire sample, high- and low-risk status groups are described in Table 1. Youth with high-risk AP-DGBI continued to evidence greater pain intensity (average pain intensity 3.6 ± 2.4 versus 2.3 ± 2.0, p-value 0.011) and functional disability (14.9 ± 10.6 versus 9.6 ± 8.1, p-value 0.014), but not anxiety (30.5 ± 17.3 versus 23.3 ± 16.3, p-value 0.066) at post-treatment, regardless of treatment allocation (see Table 1 and Fig. 1, which depicts raw, not model estimated, post-treatment means).

The results of MANCOVA modeling suggested significant independent effects of AP-DGBI risk status (F (3, 72) = 3.30, Wilks’ Λ 0.879, *p* = 0.025) in this sample. Accounting for risk status and age, there were no independent effects of treatment allocation (Table 2). Similarly, there were no interactions of AP-DGBI risk status with treatment allocation. Post hoc analyses indicate that the effects of AP-DGBI risk status were limited to pain intensity (estimated marginal mean difference post-treatment pain intensity for high versus low risk 1.47 [95% Confidence Interval (CI) 0.44, 2.50]) and functional disability (5.71 [95% CI 1.38, 10.05]) (see Table 3). There was not a significant difference in estimated marginal mean post-treatment anxiety symptoms for the high- and low- risk status groups (7.05 [-0.73, 14.84]). That is, in this sample, youth classified with high-risk AP-DGBI at baseline evidenced significantly higher pain intensity and functional disability, but not anxiety symptoms, at post-treatment compared to those with low-risk AP-DGBI regardless of treatment allocation.

**Table 2:** Multivariate analyses of covariance modeling of post-treatment outcomes (*n* = 79)

Effect	Wilks’ lambda	F (df)	*p*-value	Partial Eta squared
Treatment group	0.975	0.60(3, 72)	0.617	0.024
AP-DGBI risk group	0.879	3.30 (3, 72)	0.025	0.121
Treatment x risk	0.953	1.18 (3, 72)	0.325	0.047
Age	0.965	0.87 (3, 72)	0.461	0.035
Model	0.789	1.49 (12, 191)	0.130	0.222
Box’s M Test 26.3, F 1.3 (df1 18, df2 11,528.5), *p* = 0.150				

*AP-DGBI* abdominal pain-related disorders of gut–brain interaction, *df* degrees of freedom

**Table 3:** Estimated marginal means and mean differences with 95% confidence intervals [CI] and *P*-values

High- versus low- risk AP-DGBI		
Post-treatment pain intensity	Estimated marginal mean [95% CI]	Difference in estimated marginal means [95% CI], p-value
High-risk AP-DGBI	3.72 [2.91, 4.53]	1.47 [0.44, 2.50], 0.006
Low-risk AP-DGBI	2.25 [1.63, 2.86]	
Post-treatment functional disability		
High-risk AP-DGBI	15.15 [11.73, 18.57]	5.71 [1.38, 10.05], 0.010
Low-risk AP-DGBI	9.44 [6.85, 12.02]	
Post-treatment anxiety		
High-risk AP-DGBI	30.33 [24.19, 36.47]	7.05 [− 0.73, 14.84], 0.075
Low-risk AP-DGBI	23.28 [18.63, 27.92]	

*AP-DGBI* abdominal pain-related disorders of gut–brain interaction, CI confidence interval

## Discussion

This study examined whether the effects of ADAPT—an evidence-based, tailored CBT program—on pain intensity, functional disability, and anxiety symptoms differed for youth classified with high- versus low- risk AP-DGBI at baseline. Controlling for age, the MANCOVA model found no support for either (a) a main effect of the ADAPT intervention, or (b) an interaction of ADAPT with AP-DGBI risk status on any post-treatment outcomes in this sample. AP-DGBI risk status did, however, exert significant independent effects on post-treatment pain intensity and functional disability, suggesting the clinical validity of this approach to AP-DGBI risk categorization.

While we did not identify treatment effects of ADAPT, despite prior evidence for its efficacy among youth with AP-DGBI (Cunningham et al., 2018a, 2018b, 2021), these findings suggest that AP-DGBI risk status is a potent predictor of clinical outcomes that negates treatment effects when included in statistical models. Though earlier studies accounted for pre-treatment levels of individual symptoms, including an indicator of clinical significance across all three symptoms may approximate an underlying risk construct with significant implications for youths’ AP-DGBI severity and management (Cunningham et al., 2017; Moorman et al., 2021). Indeed, in this sample, risk status accounted for approximately 12% of the variation in post-treatment outcomes. It is possible that treatment effects may have been identified in a larger sample with greater statistical power. This may be particularly relevant to anxiety symptoms, given that, based on examination of raw post-treatment means (Fig. 1), the receipt of ADAPT appeared to widen differences between low- risk participants in the ADAPT + TAU versus TAU-alone groups.

Like related studies, risk categorization identified youth who continued to exhibit higher levels of post-treatment symptoms, suggesting the clinical validity of the construct and the importance of functional disability and pain intensity as interventional targets (Cunningham et al., 2017; Heathcote et al., 2018; Simons et al., 2015; Walker et al., 2012). Notably, high-risk-youth did not have higher post-treatment anxiety symptoms compared to low-risk peers. This may be due to the augmentation of the intervention approach (e.g., ADAPT) to specifically address anxiety symptoms. However, additional work is required in larger samples. Markedly, close to 37% of the sample was classified as high risk, reinforcing the importance of psychosocial screening in pediatric gastroenterology (Cunningham et al., 2018a, 2018b). Given that childhood and adolescent AP-DGBI is associated with negative outcomes in adulthood (e.g., chronic pain, mental health problems) (Horst et al., 2014; Shelby et al., 2013; Walker et al., 2010), identifying those most at risk for persistent and/or worsening outcomes may be of critical importance to guiding targeted, timely intervention efforts.

Yet, pathways toward the application of risk categories in clinical practice remain unclear. For example, a study of German adolescents seeking treatment for chronic pain found that while youth classified with a higher chronic pain grade were more likely to receive a recommendation for intensive inpatient management, the overall prognostic utility of a chronic pain grade (i.e., the ability to discriminate between youth referred to inpatient or outpatient treatment) was modest (area under the receiver operating curve 0.69) (Wager et al., 2013). Similarly, a study of U.S. youth failed to identify associations between risk status and treatment recommendations (Simons et al., 2015). However, such findings may be related to variations in clinical practice, or perhaps due to a lack of data supporting which treatments work best at different risk levels. Future work may be required to elucidate this possibility. For example, the results of the parent study suggest the feasibility and efficacy of a stepped approach to psychological care for youth with AP-DGBI based on functional disability and anxiety symptoms independently (Cunningham et al., 2021). It is yet unknown whether AP-DGBI risk categorization can be used to similarly guide management, but future work may test longer or more intensive interventions for high-risk youth.

It is important to note that while the current study focused on clinically elevated pain, functional disability, and anxiety given prior associations with prolonged impairment (Cunningham et al., 2017; Moorman et al., 2021), accumulating data suggest the importance of other child (stress, coping style, and sleep) and parent factors (anxiety symptoms, modeled coping behaviors) to outcomes among youth with AP-DGBI (Calvano & Warschburger, 2022; Zia et al., 2022). Such findings support the potential clinical relevance of the current risk categorization approach while emphasizing the need for future research incorporating additional child, family, and contextual factors to further refine psychosocial risk stratification in youth with AP-DGBI.

## Limitations

The generalizability of these findings is limited by a small, homogenous sample and associated low statistical power. Further work is required to replicate findings regarding the clinical validity of AP-DGBI risk categorization in a larger, more diverse sample. Second, participants were enrolled from the gastroenterology clinics associated with a single children’s hospital, potentially contributing to shared variance which may have influenced these findings in unknown ways. However, the recruitment of youth from several different outpatient clinics may mitigate this concern to some degree. Third, the dichotomous conceptualization of AP-DGBI risk categorization (e.g., high versus low) may have resulted in lost nuance, as it is possible that comparing youth with specific risk factors present may have revealed unknown variation in treatment response. However, this decision was guided by the relatively small sample size and by prior research suggesting that youth with AP-DGBI and clinical elevations across all three symptom domains were at the highest risk for persistent pain-related functional disability (Cunningham et al., 2017). Further, a brief, straightforward risk categorization system may be the most feasible to implement in busy clinical settings. Relatedly, due to the nature of the parent RCT, all of the participants in this study had greater than minimal levels of functional disability. Therefore, we were unable to compare youth with minimal or no functional disability to those with higher levels . .

## Conclusion

While there were no treatment effects of ADAPT, nor moderation by risk status in this sample, the results add to an emerging body of work suggesting the existence of a not-uncommon, high-risk AP-DGBI subgroup (Cunningham et al., 2021; Moorman et al., 2021), and the importance of psychosocial screening in this population. Though the application of risk categorization to clinical practice has yet to be clearly established, these results highlight the need for continued exploration of whether and how risk categorization impacts youths’ long-term clinical outcomes and treatment response.

## Data Availability

The data that support these findings are not available due to reasons of sensitivity and are available upon reasonable request.
